# Patient-Related Factors Associated With Long-Term Outcomes After Successful Endoscopic Balloon Dilation For Crohn’s Disease-Associated Ileo-Colic Strictures: A Systematic Review and Meta-analysis

**DOI:** 10.1093/crocol/otae041

**Published:** 2024-08-04

**Authors:** Hiram Menezes Nascimento Filho, Angelo So Taa Kum, Alexandre Moraes Bestetti, Pedro Henrique Veras Ayres da Silva, Megui Marilia Mansilla Gallegos, Adérson Omar Mourão Cintra Damião, Udayakumar Navaneethan, Eduardo Guimarães Hourneaux de Moura

**Affiliations:** Gastrointestinal Endoscopy Unit, Hospital das Clínicas da Faculdade de Medicina da Universidade de São Paulo, São Paulo, Brazil; Gastrointestinal Endoscopy Unit, Hospital das Clínicas da Faculdade de Medicina da Universidade de São Paulo, São Paulo, Brazil; Gastrointestinal Endoscopy Unit, Hospital das Clínicas da Faculdade de Medicina da Universidade de São Paulo, São Paulo, Brazil; Gastrointestinal Endoscopy Unit, Hospital das Clínicas da Faculdade de Medicina da Universidade de São Paulo, São Paulo, Brazil; Gastrointestinal Endoscopy Unit, Hospital das Clínicas da Faculdade de Medicina da Universidade de São Paulo, São Paulo, Brazil; Department of Gastroenterology and Hepatology, Hospital das Clínicas da Faculdade de Medicina da Universidade de São Paulo, São Paulo, Brazil; Orlando Health Digestive Health Institute Center for Advanced Endoscopy, Research and Education, Orlando, USA; Gastrointestinal Endoscopy Unit, Hospital das Clínicas da Faculdade de Medicina da Universidade de São Paulo, São Paulo, Brazil

**Keywords:** Crohn’s disease, strictures, endoscopic balloon dilation

## Abstract

**Background:**

Successful Crohn’s disease (CD) therapy relies on timely and precise management strategies. Endoscopic balloon dilation (EBD) has been applied as a first-line treatment for symptomatic CD-associated strictures due to its minimally invasive nature and the possibility of preserving intestinal length.

**Objective:**

The aim of the present study was to determine patient-related predictive factors associated with the need for surgery for CD-associated ileocolic strictures after technically successful EBD.

**Methods:**

All original studies published before December 2023 that reported the outcomes of patients treated with EBD for ileocolic strictures secondary to CD and described follow-up for at least 1 year were included. The difference in risk of needing surgery was calculated for 8 different patient characteristics (Sex, smoking habit, previous surgery, biologic therapy, steroids, immunosuppressors, nature of the stricture, and endoscopic disease activity).

**Results:**

There were significant differences in the risk of needing surgery after EBD among patients who underwent surgery and patients without a history of surgery (RD: −0.20 [−0.31, −0.08]), patients with endoscopic mucosal activity and patients in remission at the time of EBD (RD: 0.19 [0.04, 0.34]), patients using biologics at the time of EBD and patients not using biologics (RD: −0.09 [−0.16, −0.03]), and patients using steroids and those not using steroids at the time of EBD (RD: 0.16 [0.07, 0.26]).

**Conclusions:**

The use of biologics and endoscopic disease remission at the time of EBD were protective factors against the need for surgery. No previous surgery or use of steroids at the time of EBD was associated with the need for surgery during follow-up.

## Graphical Abstract

**Graphical Abstract:**
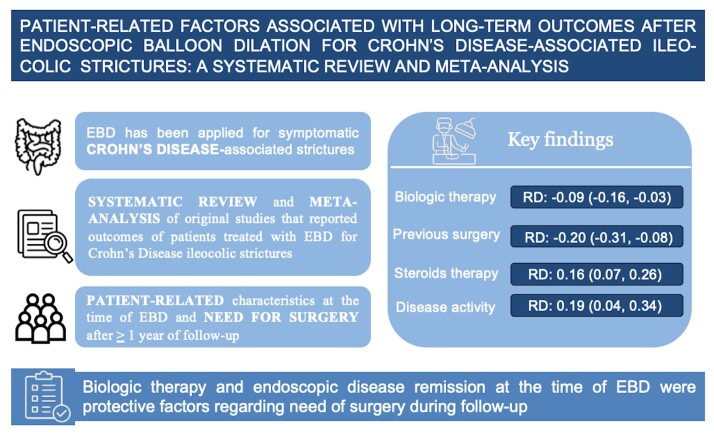

**Key Message**

- What is already known?

Endoscopic balloon dilation is a safe and efficacious therapy for CD-associated strictures. A short period of obstructive symptoms and noncomplicated and short strictures are associated with endoscopic therapy success.

- What is new here?

Patients suffering from CD who are taking biologics and who experience endoscopic remission at the time of endoscopic balloon dilation have a decreased need for surgery during follow-up.

- How can this study help patient care?

The findings from the present study may help clinicians involved in inflammatory bowel disease care provide assertive therapy to patients with stricturing CD.

## Introduction

Crohn’s disease (CD) is a chronic, relapsing-remitting, immune-mediated condition that can affect any region of the intestine, often through transmural inflammation.^1^ The overall incidence of inflammatory bowel diseases (IBD) continues to increase. Over the last 2 decades, the incidence of CD in North America and Europe has stabilized, whereas traditionally low-prevalent areas (especially low-income countries) have experienced a significant increase. These dynamic changes have critical implications for health systems and local economies.^2^

Medical advancements have been made in treating IBD through understanding its pathogenesis, host immune response, and genetic signature.^3–5^ Despite the growing knowledge, successful CD therapy is dependent on timely and precise management strategies.^6^ These strategies rely on disease course and prognosis as cornerstones for therapeutic decisions and frequently work as “one-size-fits-all” approaches. To apply an individualized approach, matching the right patient to the right treatment, biomarkers, and disease outcomes need to be investigated.

Population-based cohort studies estimate that up to one-quarter of CD patients have a stricturing phenotype.^7,8^ Patients with CD-associated strictures are at greater risk for the development of intestinal obstruction, fistulae, and abscesses, which are common indications for surgery. Even though the risk of intestinal surgery among patients with IBD has decreased over the last 3 decades, it remains significantly high (33% and 46% at 5 and 10 years after diagnosis of CD, respectively).^9^ In addition to the risk of postoperative morbidity and mortality, multiple surgeries predispose patients to the risk of short bowel syndrome, which makes bowel-conserving strategies appealing.

Recently, enthusiasm has been expressed in the field of CD therapy, including modern drugs and surgical bowel-preserving strategies. Endoscopic balloon dilation (EBD) has been applied as a first-line treatment for symptomatic CD-associated strictures due to its minimally invasive nature and the possibility of preserving intestinal length. A previous meta-analysis confirmed the safety and efficacy of EBD for treating CD-associated strictures.^10,11^ Indeed, EBD significantly improves the symptomatic response and prevents surgical delay, serving as a bridge between medical and surgical therapy.

After technically successful EBD, approximately half of CD patients need surgical intervention for treated strictures.^12^ Technical issues have been largely studied, and prognostic factors associated with less surgical intervention have been recognized.^10,13^ Little is known about patient-related predictive factors associated with the long-term outcomes of EBD. The aim of the present study was to determine patient-related predictive factors associated with the need for surgery for CD-associated ileocolic strictures after technically successful EBD.

## Materials and Methods

### Protocol and Registration

This systematic review and meta-analysis of prognostic factor studies followed the guidelines proposed by Riley et al.^14^ and Moons et al.^15^ The study protocol was registered in the International Prospective Register of Systematic Reviews (PROSPERO) database (https://www.crd.york.ac.uk/prospero/) under file number CRD42024498998. This study was approved by the Ethics Committee of Hospital das Clínicas, Faculty of Medicine at the University of São Paulo.

### Search Strategy

An online search in accordance with the Preferred Reporting Items for Systematic Reviews and Meta-Analyses (PRISMA) guidelines^16^ was conducted using the following databases: MEDLINE and EMBASE. A 3-step search strategy was employed. Initially, a limited search was performed using PubMed to identify keywords and index terms contained in the title or abstract. The second step involved an extensive search using all identified keywords and extensive terms. For the third step, the following mesh terms were applied in the mentioned databases: Crohn’s Enteritis OR Regional Enteritis OR Crohn’s Disease OR Crohns Disease OR Inflammatory Bowel Disease 1 OR Enteritis, Granulomatous OR Granulomatous Enteritis OR Enteritis, Regional OR Ileocolitis OR Colitis, Granulomatous OR Granulomatous Colitis OR Ileitis, Terminal OR Terminal Ileitis OR Ileitis, Regional OR Regional Ileitides OR Regional Ileitis) AND (Constrictions, Pathologic OR Pathologic Constrictions OR Stricture OR Strictures OR Stenosis OR Stenoses OR Constriction, Pathological OR Pathological Constriction OR Pathologic Constriction). The final step was a manual search of reference lists and bibliographies from previously retrieved studies to identify further relevant trials.

### Eligibility and Exclusion Criteria

All original studies published before December 2023 that reported outcomes of EBD for ileal or colonic strictures secondary to CD in patients aged ≥16 years and described follow-up for at least 1 year post-EBD were included in the review. Articles were included according to their type, randomized controlled trials, cohort studies, and case–control studies, and their abstracts and full texts were available. The following exclusion criteria were applied: <16 years of age, CD-associated upper gastrointestinal strictures (esophagus, stomach, or duodenum), Ulcerative Colitis (UC) or indeterminate colitis, intralesional injection of anti-TNF alpha, case series, case reports, conference proceedings, and abstracts only.

### Data Collection Process

The first reviewer (HM) screened for titles and abstracts identified in the search strategy. The articles were then evaluated by 2 reviewers (HM and AK) according to the eligibility criteria outlined above. Discrepancies were resolved by consensus between the 2 reviewers. The data from the selected studies were extracted by the first reviewer, after which the data were further checked by the second reviewer. The extracted data were entered into the Excel (Microsoft software) database.

### Data Items

The following data were extracted: Study demographics (year and country of publication, study design, sample size, and follow-up period), patient characteristics at the EBD (age, smoking status, use of immunosuppressants, biologics and steroids, and history of previous surgery), the nature of the stricture and endoscopic activity status.

### Risk of Bias in Individual Studies and Quality of Evidence

The quality of the studies was assessed by using the Quality in Prognosis Studies (QUIPS) tool. The quality of the studies was evaluated by examining 6 corresponding domains: Participation, attrition, prognostic factor measurement, outcome measurement, confounding, statistical analysis, and reporting.^17^

### Summary Measures

According to the Global Interventional Inflammatory Bowel Disease Group, noncomplicated strictures were defined as the absence of abscess or fistula. A symptomatic response was defined as an obstructive symptom-free outcome at the end of follow-up. The technical response was defined by the passage of the scope following EBD, and long-term efficacy was defined as surgery-free survival for one year after any endoscopic treatment.

### Statistical Analysis and Risk of Bias Across Studies

The software Review Manager ([RevMan] Version 5.4.1 The Cochrane Collaboration) was used. Continuous numerical data are expressed as the means and standard deviations (SDs). A per-study analysis was used to assess pooled event rates across studies. The risk difference (RD) was calculated for each independent variable with a 95% confidence interval (CI). If a study provided medians and interquartile ranges, the means and SDs were calculated based on the McGrath method.^18^ Heterogeneity among studies was assessed using the I^2^ index introduced by the Higgins method^19^ and visually visualized with forest plots. In the case of high heterogeneity across studies (*I*^*2*^ > 50%), a random effects model was also used for the corresponding variable. The results are expressed as forest plots and statistical data.

## Results

### Search Strategy

**Figure 1.**
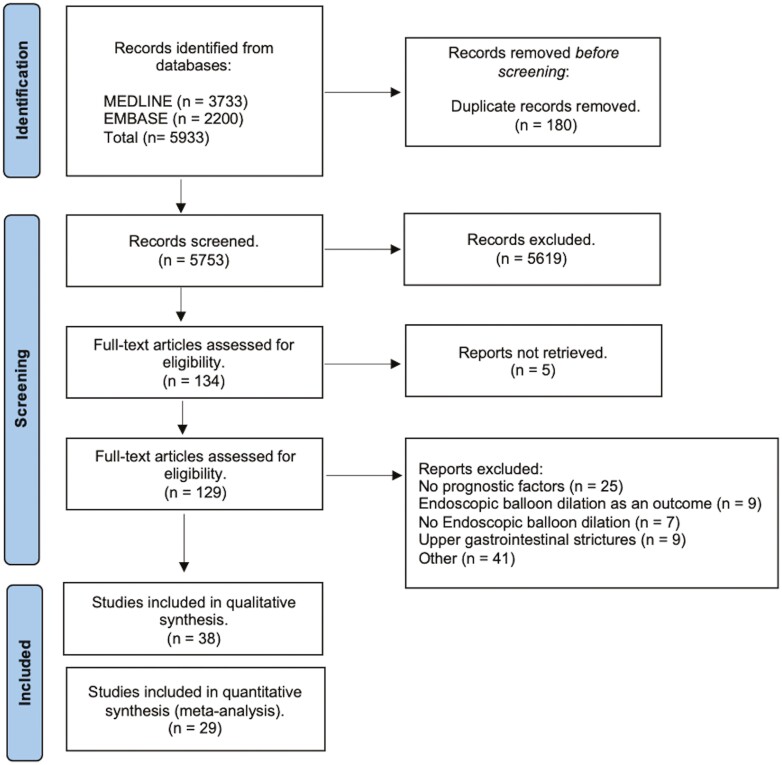
PRISMA flow chart.

The initial search retrieved 5933 records from MEDLINE and EMBASE. Screening of titles and abstracts identified 134 articles for full-text review. A total of 38 articles fulfilled the eligibility criteria. Finally, 29 studies were included in quantitative analysis (Figure 1).

### Study Characteristics

**Figure 2.**
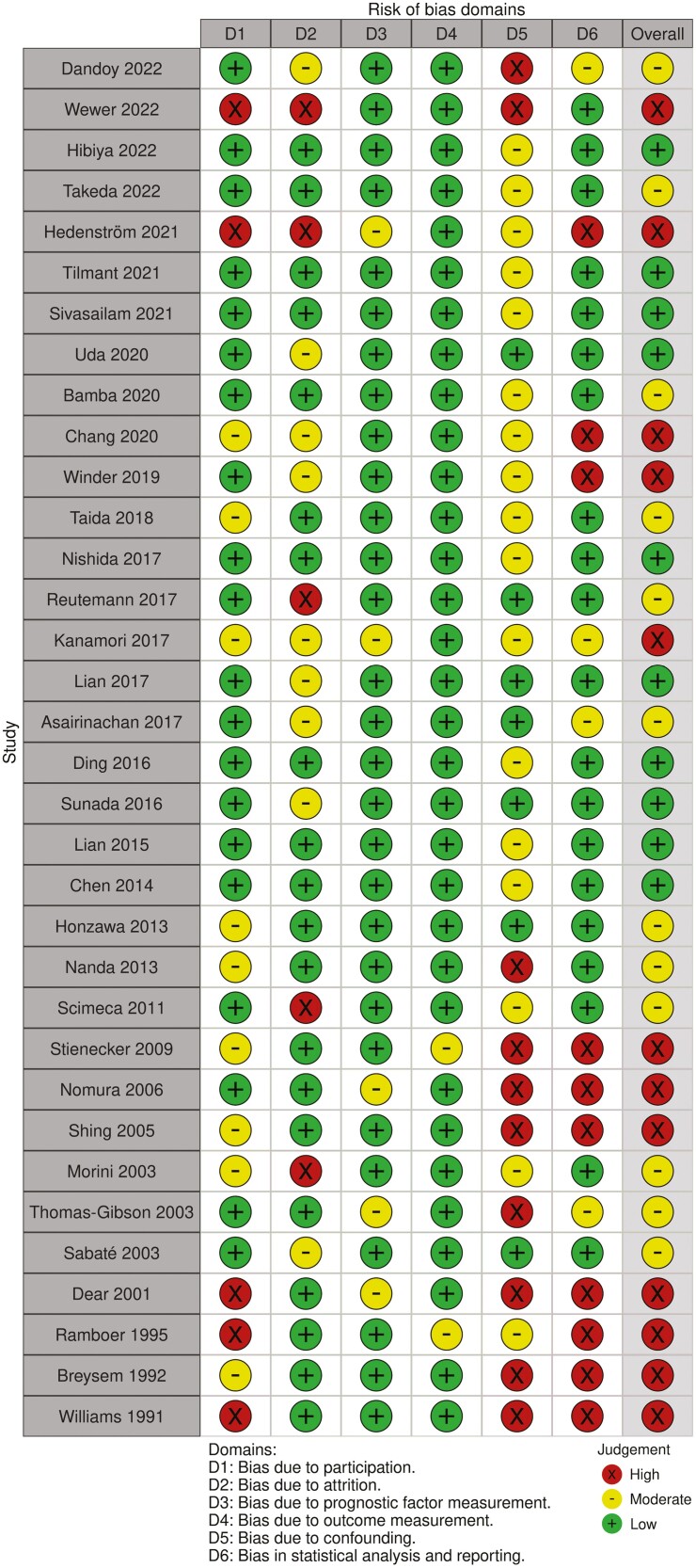
Quality in Prognosis Studies (QUIPS) tool for risk of bias assessment.

Data from 29 observational studies, comprising 1632 patients, was processed for quantitative analysis. Twenty-five studies reported mean follow-up; 20 studies reported mean age and mean disease duration at first EBD; only 1 study did not report any data regarding demographic characteristics (Table 1). The risk of bias in individual studies is shown in Figure 2.

**Table 1.** Demographic characteristics of studies included in meta-analysis.

Study	Region	Sample size	Follow-up (m)	Age at intervention (y)	Disease duration (y)
Dandoy 2022^20^	Belgium	94	45.5 ± 28.9	46.9 ± 4.6	
Wewer 2022^21^	Denmark	90			
Hibiya 2022^22^	Japan	98			4.6
Takeda 2022^23^	Japan	72	50.9 ± 10.3	36.9 ± 2.9	13.1 ± 2.3
Tilmant 2021^24^	France	57	53.3 ± 16.7	36.6 ± 3.7	12.9 ± 3.3
Sivasailam 2021^25^	United States	99	64.8 ± 45.1	37.1 ± 3.6	14.1
Bamba 2020^26^	Japan	100		34.9 ± 3	5.4 + 2.2
Chang 2020^27^	Taiwan	26	42.5 ± 13.9	38.7 ± 14.8	6.3 ± 5.4
Winder 2019^28^	Israel	64		43.3 ± 14.2	15.8 ± 9.0
Taida 2018^29^	Japan	26	29.4 ± 24.9	39.3 ± 13.8	8.1 ± 7.6
Nishida 2017^30^	Japan	37	27.8 ± 13.6	35.0 ± 1.4	10 ± 2
Reutemann 2017^31^	United States	135	42.8 ± 10.6		
Kanamori 2017^32^	Japan	30	13.4 ± 9.8	36.9 ± 10.7	13.1 ± 8.8
Asairinachan 2017^33^	Australia	47	37.9 ± 8.1	39.7 ± 3.8	13 ± 3.2
Ding 2016^34^	England	54	71.9 ± 10.3	54 ± 3.5	23.9 ± 2.9
Sunada 2016^35^	Japan	85	45.5 ± 28.9		
Lian 2015^36^	United States	185	46.8 ± 34.8		17.6 ± 10.7
Chen 2014^37^	United States	60	50.0 ± 31.1	43.9 ± 12.4	
Honzawa 2013^38^	Japan	25	46.3 ± 23.9	37 ± 8	12
Nanda 2013^39^	Ireland	31	41 ± 35.7	44 ± 13	16.2 ± 3.4
Scimeca 2011^40^	Italy	37	27.4 ± 13.9		
Stienecker 2009^41^	Germany	25	82.3 ± 16.3		12.6 ± 7.5
Nomura 2006^42^	Japan	16	40.7 ± 18.7	29.6 ± 6.2	9.5 ± 6.5
Singh 2005^43^	United States	14	21.9 ± 13.2	43 ± 17.4	
Sabaté 2003^44^	France	38	28.8 ± 24	33.7 ± 9.6	10.2 ± 6.9
Morini 2003^45^	Italy	34	63.7 ± 44.6		
Dear 2001^46^	England	22	45.6 ± 22.5		
Ramboer 1995^47^	Belgium	13	44.8 ± 19.1	44.3 ± 12.5	20.1 ± 13.2
Breysem 1992^48^	Belgium	18	18.1 ± 10.4	40.9	14.9
Total		1632	41	44.5	11.7

### Predictive Factors Associated With the Need for Surgery During Follow-up After EBD

#### Patient characteristics

##### Gender

**Figure 3.**
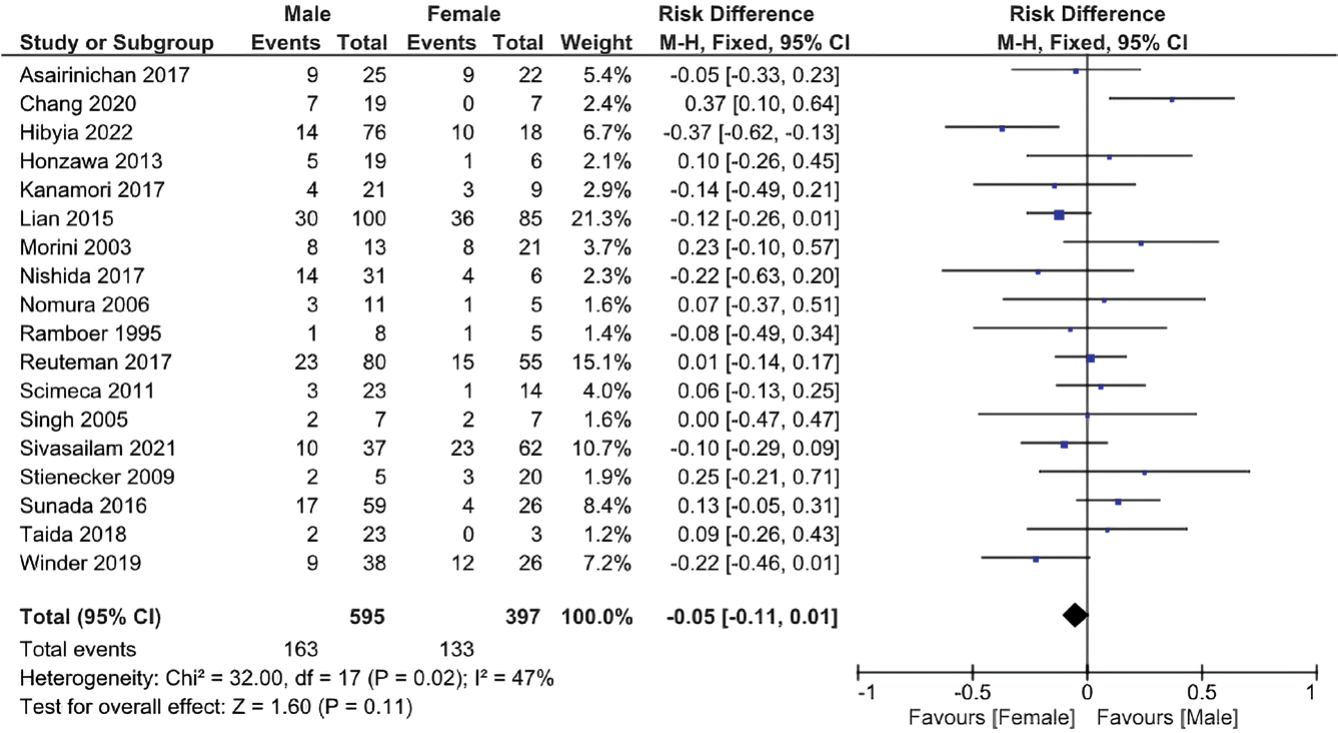
Effect of gender on the need for surgery after EBD. M-H, Mantel-Haenszel; CI, confidence interval.

There was no statistically significant difference related to the need for surgery after EBD between genders (RD: −0.05 [−0.11, 0.01]). Figure 3.

##### Smoking habit

**Figure 4.**
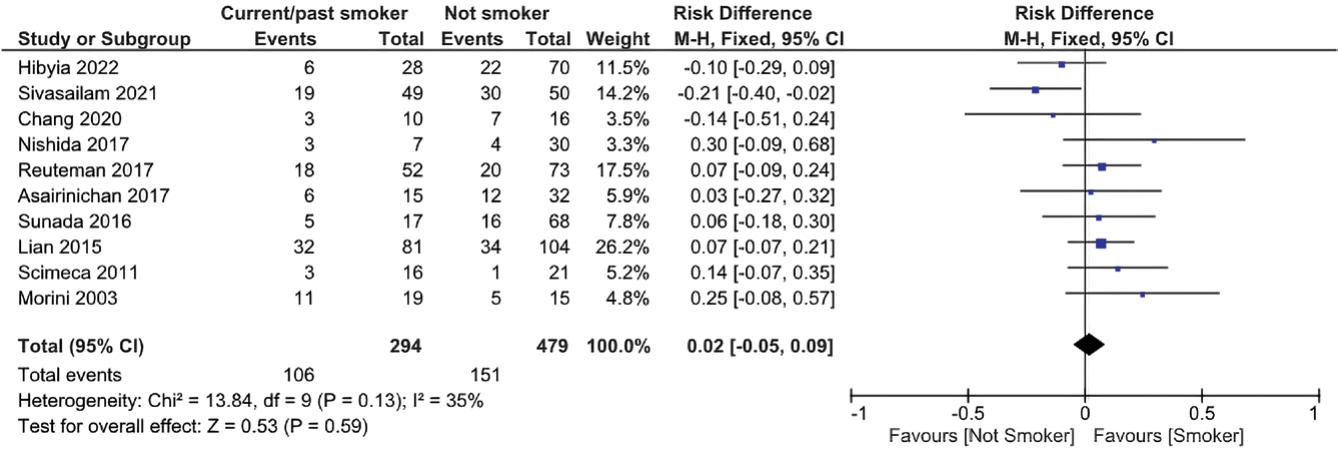
Effect of smoking habit on the need for surgery after EBD. M-H: Mantel-Haenszel; CI, confidence interval.

There was no statistically significant difference related to the need for surgery after EBD between patients who were never smokers and patients who were past–current smokers (RD: 0.02 [−0.05, 0.09]). Figure 4.

##### Previous surgery

**Figure 5.**
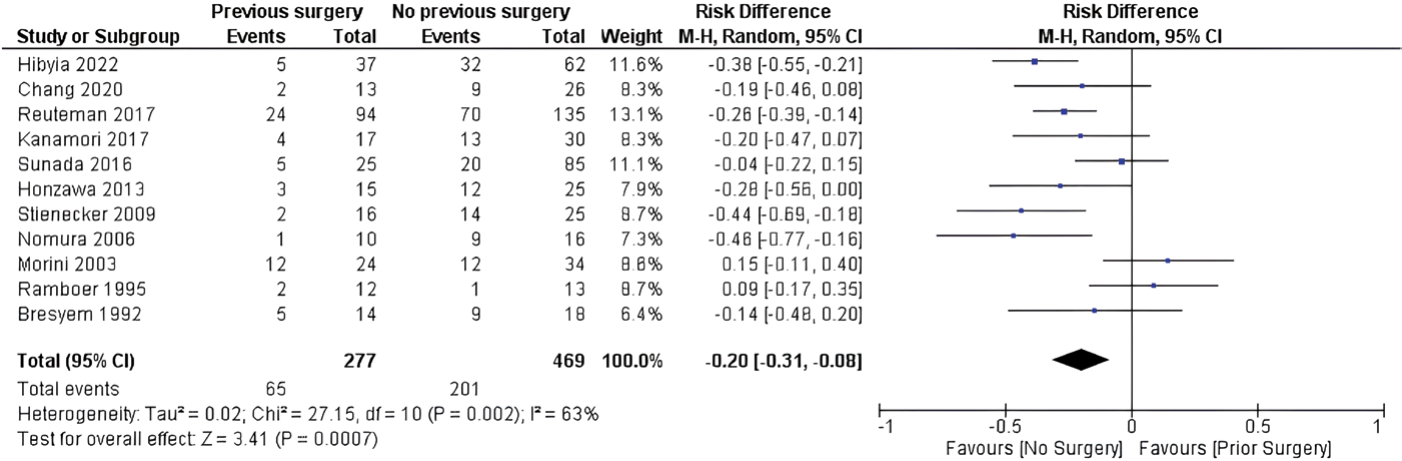
Effect of previous CD-related surgery on the need for surgery after EBD. M-H, Mantel-Haenszel; CI, confidence interval.

There was a statistically significant difference related to the need for surgery after EBD between patients who did not undergo past surgery and patients with previous surgery related to CD (RD: −0.20 [−0.31, −0.08] *P* < .001 I^2^ = 63%). Figure 5.

#### Medical treatment

##### Biologic therapy

**Figure 6.**
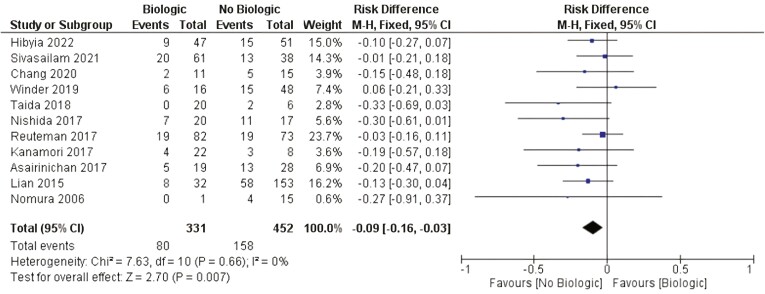
Effect of biologic treatment on the need for surgery after EBD. M-H, Mantel-Haenszel; CI, confidence interval.

There was a statistically significant difference related to the need for surgery after EBD between patients who were using biologics and those patients not using biologics (RD: −0.09 [−0.16, −0.03] *P* < .01 I^2^ = 0). Figure 6.

##### Steroids therapy

**Figure 7.**
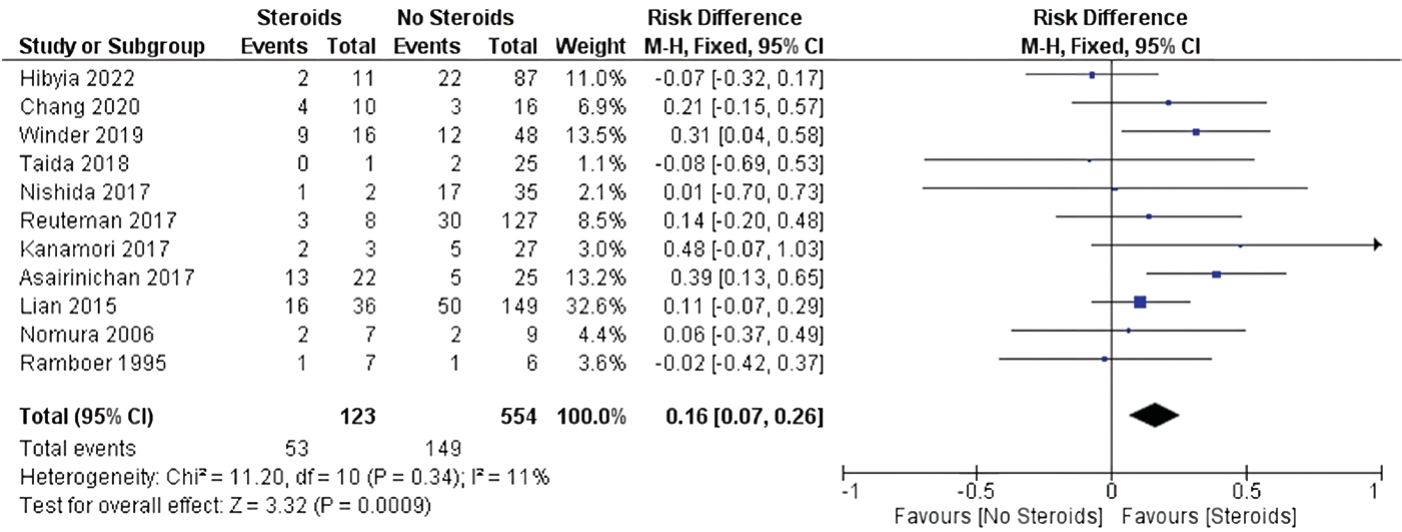
Effect of steroids on the need for surgery after EBD. M-H, Mantel-Haenszel; CI, confidence interval.

There was a significant statistical difference related to the need for surgery between patients who were using steroids and patients not using steroids after EBD (RD: 0.16 [0.07, 0.26] *P* < .001 I^2^ = 0). Figure 7.

##### Immunosuppressor therapy

**Figure 8.**
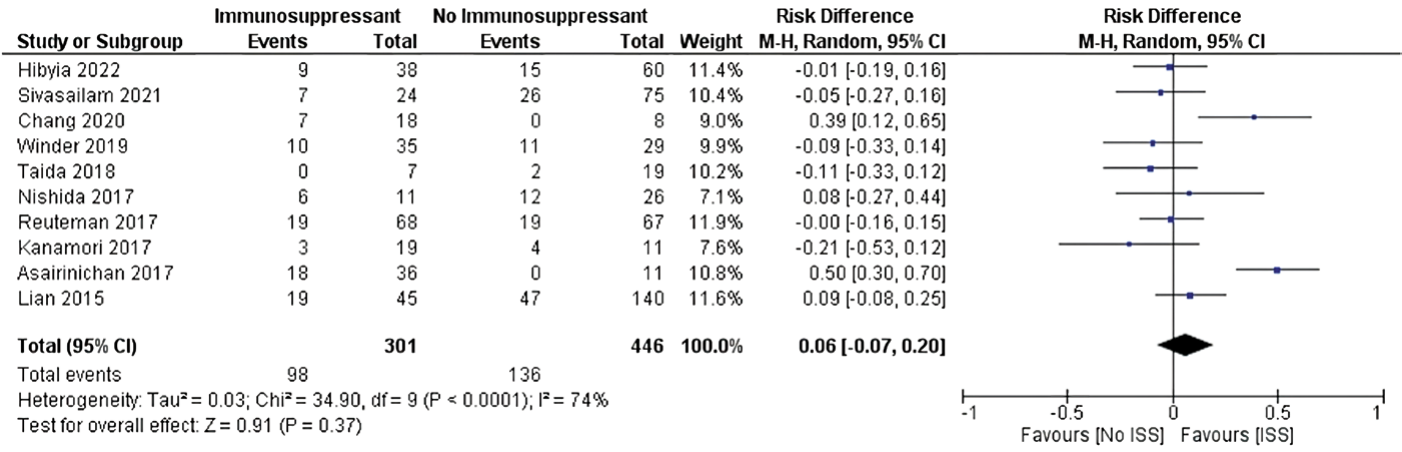
Effect of immunosuppressants on the need for surgery after EBD. M-H, Mantel-Haenszel; CI, confidence interval.

There was no statistically significant difference related to the need for surgery between patients who were using immunosuppressant agents (ISS) and patients who were not using ISS after EBD (RD: 0.06 [−0.07, 0.20]). Figure 8.

#### Strictures characteristics

##### Nature of the stricture

**Figure 9.**
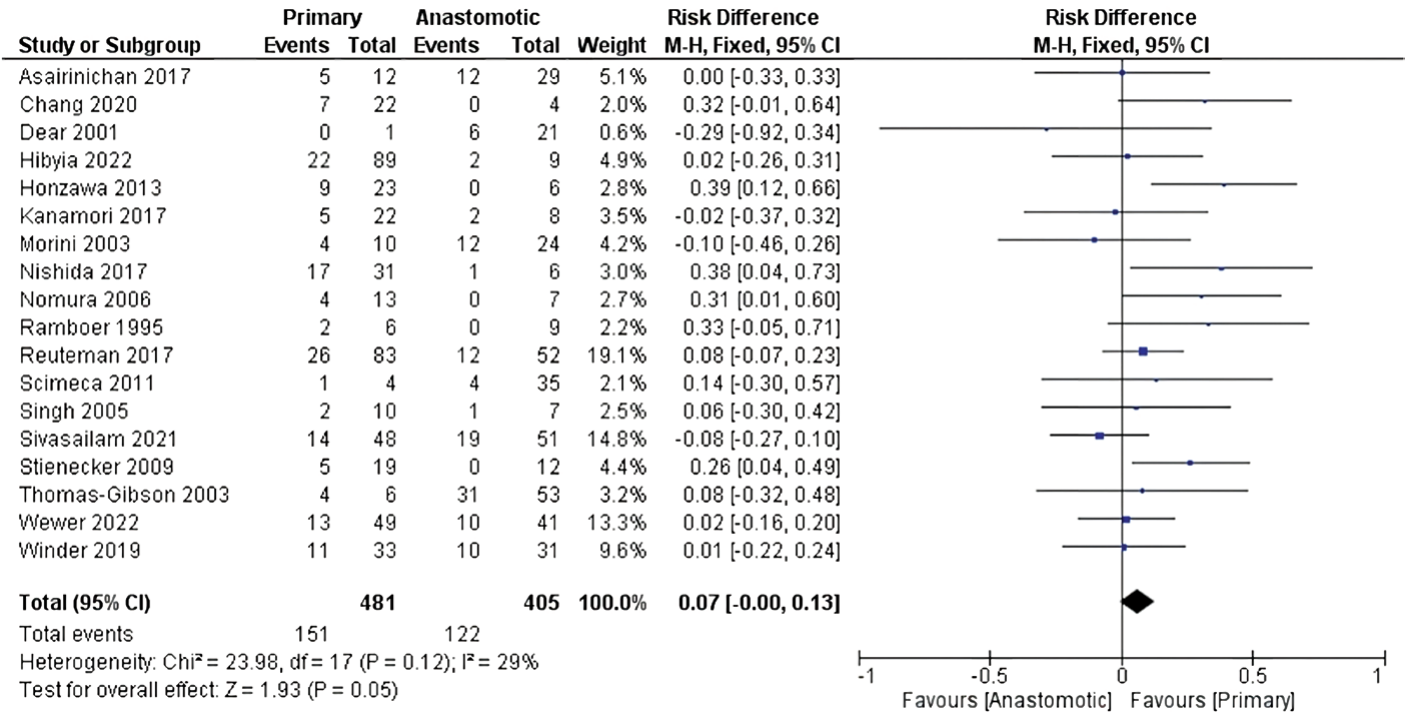
Effect of the nature of the stricture on the need for surgery after EBD. M-H, Mantel-Haenszel; CI, confidence interval.

There was no statistically significant difference related to the need for surgery between patients who presented primary strictures and patients who presented anastomotic strictures after EBD (RD: 0.07 [−0.00, 0.13]). Figure 9.

##### Endoscopic disease activity

**Figure 10.**
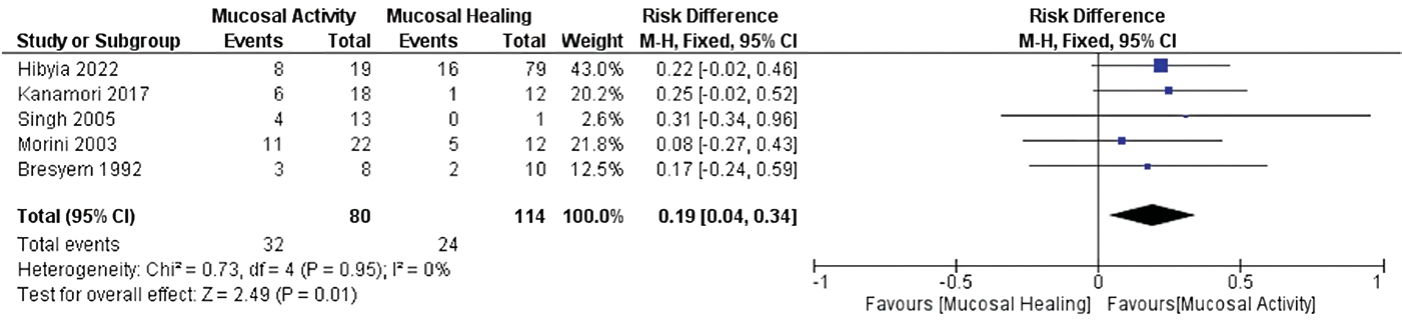
Effect of endoscopic disease activity on the need for surgery after EBD. M-H, Mantel-Haenszel; CI, confidence interval.

There was a statistically significant difference related to the need for surgery between patients who presented endoscopic mucosal activity and those in endoscopic remission after EBD (RD: 0.19 [0.04, 0.34] *P* < .05 I^2^ = 0). Figure 10.

## Discussion

To the best of our knowledge, we present the first systematic review and meta-analysis in which the main investigation on EBD involved evaluating patients via pathology. Since the first report of EBD for CD-associated strictures,^49^ many studies regarding this technique have been published. Most of them presented data on stricture and technique features (stricture length, fibrotic and inflammatory components, and balloon diameter); however, only a few described patient-related characteristics and their influence on EBD outcomes. In light of the available data, most of the studies included in our study respected the technical aspects associated with technical success.

Although not completely understood, sex differences impact the pathogenesis, disease course, and response to therapy in patients with CD. Females experience earlier disease onset, extraintestinal manifestations (EIMs), and nonfistulizing perianal disease more frequently than males.^50,51^ Sex hormones and sex-dependent epigenetic changes may significantly influence the observed differences. Moreover, nonbiological features regarding access to care and lifetime exposure may also be relevant.^52^ Our study did not reveal any difference between males and females regarding the need for surgery after EBD.

Smoking habits are associated with the risk of developing CD, disease progression, severity, and disease flares.^53^ It is postulated that tobacco (nicotine and other chemical constituents) alters the gut microbiota and gut permeability and triggers an aberrant local immune response in a dose-dependent manner.^54^ Even though smoking is a recognizable factor associated with poor prognosis in the course of CD, our study did not reveal any difference between patients who were never smokers and patients who were past–current smokers regarding the need for surgery after EBD.

It is largely debated whether multiple CD-related surgeries are risk factors for worse outcomes and disease recurrence. Surgery is not curative, and more than half of patients will experience disease recurrence adjacent to the anastomosis within 1 to 5 years postsurgery.^55^ Beyond the disease’s natural course, primary surgery is frequently performed via a laparoscopic approach under inflammatory and noncomplicated conditions, which is different from reoperation, the moment at which surgeons frequently face complex adhesions, altered anatomy, and hidden perforating disease with abscesses and fistulas.^56^ In our study, we evaluated surgical history related to CD. A significant difference in the risk of needing surgery after EBD was shown among patients who did not undergo previous surgery. A possible explanation for this finding may be the delay in surgery in many instances of CD. In this situation, the inflammatory process is replaced by fibrosis of variable length—sometimes >5–6 cm—in the affected intestine, which may reduce the chances of successful treatment with EBD.

Stricture formation results from an inflamed intestine and is mainly driven by the depth and chronicity of inflammation, leading to architectural changes, mural thickening, loss of mucosal compliance, and contracture.^57^ There is no medical therapy available for treating CD fibrosis. Therefore, symptomatic patients who fail to respond to medical therapy often require surgical intervention. Biologics agents and immunosuppressants, mostly infliximab, might act as powerful anti-inflammatory agents when inflammation and edema coexist with fibrosis in CD-associated strictures. Reducing cell infiltration and edema in the bowel wall leads to luminal narrowing and obstructive symptom improvement.^58^ Our study revealed that patients who used biologics at the time of EBD had significant differences in risk, protecting them from the need for surgery. This effect was not observed for patients receiving immunosuppressants, such as azathioprine. Although not completely understood, biologics have positive, long-lasting effects on patients with stricturing CD. Among the 11 studies included in the analysis of biologic use at the time of EBD, only one differentiated the use of anti-TNF-alpha and other biologics.

Systemic corticosteroids have been used to treat CD for more than 60 years. These substances exert their anti-inflammatory effects on IBD mainly by inhibiting the IL-1, IL-6, and TNF-alpha pathways.^59^ Since the publication of the Cooperative Crohn’s Disease Study (NCCDS) and European Cooperative Crohn’s Disease Study (ECCDS), first-generation corticosteroids (prednisone, prednisolone, and methylprednisolone) have become well-established agents for the induction of remission in patients with moderate-to-severe CD.^60,61^ Despite the temporary beneficial effects of these agents, a considerable group of patients experience disease relapse and steroid resistance during the disease course. Most of these patients are young at diagnosis, have a colonic or anoperineal location of disease, and are former–current smokers.^62^ Indeed, long-term use of corticosteroids is associated with serious adverse events, including hyperglycemia, hypertension, bone metabolic disorders, central nervous system disorders, growth retardation, and increased risk of infections.^63^ Despite the wide use of corticosteroids in this population, only one study included in the quantitative analysis described the dose and route of administration. In agreement with the expected more aggressive phenotype of CD steroid-dependent patients, our study revealed RDs favoring the need for surgery after EBD for individuals using steroids.

CD-associated strictures are classified as primary or postsurgical (anastomotic). Strictures might develop at any segment of the gastrointestinal tract, whereas they predominate in the terminal ileum and ileocolonic anastomosis.^55^ Primary strictures develop as a consequence of tissue damage caused by disease activity. Anastomotic strictures might develop because of technical failure, patient-related perioperative factors, or postoperative disease recurrence.^64^ A systematic review and meta-analysis revealed no difference in clinical success or technical failure between primary and anastomotic stricture patients treated with EBD.^65^ Our study corroborates the lack of significant differences regarding the need for surgery after EBD for primary or anastomotic stricture, provided that technical issues are met.

Historically, both clinical and endoscopic disease activity are associated with poor long-term outcomes, including penetrating complications and surgery.^66^ Since the introduction of the anti-TNF alpha agents and, subsequently, the anti-IL12/23 and anti-integrin agents, mucosal healing has become a feasible treatment target, the achievement of which is associated with a reduced risk of relapse, hospitalization, and surgery.^67,68^ Our results confirm the observation that endoscopic mucosal activity at the time of EBD is associated with the need for surgery during follow-up.

Despite the use of careful methodology, our review has several limitations. All the included studies were observational, and most of them involved retrospective cohorts. As expected in prognostic factor research, there is great variability among primary studies related to study design, number of patients, follow-up duration, outcomes, and measured prognostic factors. Our analysis provided information on only unadjusted prognostic effects given the lack of available data. Moreover, it was impossible to discriminate medical treatment according to biological classes, to determine the dose of corticosteroids associated with surgical intervention, to evaluate patients who stopped smoking during follow-up and to evaluate the number of previous surgeries. Finally, studies have been conducted before the era of biological therapy, a therapeutic approach that certainly impacts the natural history of CD.

In summary, our study must be carefully interpreted. Even though the information provided is prone to bias, it is essential to note that most evidence of prognostic factors is generated from low-evidence studies. In addition, EBD and surgery are complementary approaches instead of concurrent modalities, and patients must be selected according to their individual clinical presentation. Our study does not intend to change the current therapy for CD. The tool targets gathering knowledge that may support multidisciplinary evaluation considering the disease course and patient background. Finally, the design of prospective studies is encouraged to validate the presented findings.

## Conclusion

The use of biologics and evidence of endoscopic disease remission at the time of EBD were protective factors against the need for surgery during follow-up. The absence of previous surgery and the use of steroids at the time of EBD were associated with the need for surgery during follow-up. Gender, smoking habit, therapy with immunosuppressants, and the nature of the stricture were not significantly related to the need for surgery after EBD. Awareness of prognostic factors associated with surgery deferral and increased need for surgery after EBD helps in the decision of multidisciplinary teams. The information provided supports the selection of patients who would benefit more from endoscopic treatment and who might benefit from surgery as a first-line approach for symptomatic strictures.

## Supplementary Material

otae041_suppl_Supplementary_Data

## Data Availability

The authors confirm that the data supporting the findings of this study are available within its supplementary materials.
